# Heterogeneous matrix stiffness regulates the cancer stem-like cell phenotype in hepatocellular carcinoma

**DOI:** 10.1186/s12967-022-03778-w

**Published:** 2022-12-03

**Authors:** Jiayun Wei, Jia Yao, Chendong Yang, Yongcui Mao, Dan Zhu, Ye Xie, Pinyan Liu, Mengchao Yan, Longfei Ren, Yan Lin, Qiuxia Zheng, Xun Li

**Affiliations:** 1 First Clinical Medical College, Lanzhou University, Lanzhou, 730000 China; 2 Key Laboratory of Biotherapy and Regenerative Medicine, First Hospital of Lanzhou University, Lanzhou University, 1st West Donggang Road, Chengguan District, Lanzhou, 730000 China; 3 General Surgery Department, First Hospital of Lanzhou University, Lanzhou University, Lanzhou, 730000 China; 4 Civil Engineering and Mechanics College, Lanzhou University, Lanzhou, 730000 China

**Keywords:** Matrix stiffness, Cancer cell stemness, Heterogeneity, Hepatocellular carcinoma, Yes-associated protein

## Abstract

**Background:**

Solid tumors are stiffer than their surrounding normal tissues; however, their interior stiffness is not uniform. Under certain conditions, cancer cells can acquire stem-like phenotypes. However, it remains unclear how the heterogeneous physical microenvironment affects stemness expression in cancer cells. Here, we aimed to evaluate matrix stiffness heterogeneity in hepatocellular carcinoma (HCC) tissues and to explore the regulation effect of the tumor microenvironment on stem-like phenotypic changes through mechanical transduction.

**Methods:**

First, we used atomic force microscopy (AFM) to evaluate the elastic modulus of HCC tissues. We then used hydrogel with adjustable stiffness to investigate the effect of matrix stiffness on the stem-like phenotype expression of HCC cells. Moreover, cells cultured on hydrogel with different stiffness were subjected to morphology, real-time PCR, western blotting, and immunofluorescence analyses to explore the mechanotransduction pathway. Finally, animal models were used to validate in vitro results.

**Results:**

AFM results confirmed the heterogenous matrix stiffness in HCC tissue. Cancer cells adhered to hydrogel with varying stiffness (1.10 ± 0.34 kPa, 4.47 ± 1.19 kPa, and 10.61 kPa) exhibited different cellular and cytoskeleton morphology. Higher matrix stiffness promoted the stem-like phenotype expression and reduced sorafenib-induced apoptosis. In contrast, lower stiffness induced the expression of proliferation-related protein Ki67. Moreover, mechanical signals were transmitted into cells through the integrin–yes-associated protein (YAP) pathway. Higher matrix stiffness did not affect YAP expression, however, reduced the proportion of phosphorylated YAP, promoted YAP nuclear translocation, and regulated gene transcription. Finally, application of ATN-161 (integrin inhibitor) and verteporfin (YAP inhibitor) effectively blocked the stem-like phenotype expression regulated by matrix stiffness.

**Conclusions:**

Our experiments provide new insights into the interaction between matrix stiffness, cancer cell stemness, and heterogeneity, while also providing a novel HCC therapeutic strategy.

**Supplementary Information:**

The online version contains supplementary material available at 10.1186/s12967-022-03778-w.

## Background

Primary liver cancer is the sixth most common cancer worldwide and the third leading cause of cancer-related deaths, with hepatocellular carcinoma (HCC) accounting for 75–85% of cases [1]. Although considerable progress has been made in the diagnosis and treatment of HCC over the past few decades, treatment efficacy remains unsatisfactory [2]. Common treatment resistance and relapse are driven primarily by the inherent heterogeneity within cancer, which allows drugs to eliminate some, but not all, malignant cells [3].

Cancer stem cell (CSC) models have been used to explore cellular heterogeneity. CSCs, also known as tumor-initiating cells (T-ICs), are present in solid tumors and constitute a small fraction of cancer cells [4]. CSCs generate cell heterogeneity by setting a differentiation hierarchy in tumors, resulting in a range of different cell types [5]. Hepatic CSCs are considered responsible for the heterogeneous and hierarchical organization of HCC [6–8]. Under certain conditions, CSCs and non-CSCs can transform into each other; that is, terminally differentiated cells can reverse differentiation and obtain characteristics of CSCs [9]. For example, the inflammatory tumor microenvironment (TME) promotes cancer cell dedifferentiation into CSCs, and the paracrine effects of stromal cells can also modulate the cancer stem-like phenotype [10].

The TME, the “soil” of cancer cells, is highly heterogeneous; previous studies have focused on the associated cellular components, including cancer-associated fibroblasts (CAFs) and immune cells [11–13]. As the role of the physical microenvironment in tumors has become better understood, stiffness, a commonly altered feature in solid tumors, has gained increasing attention. Stiffness is the ability of a material to resist elastic deformation under force, typically measured by the elastic modulus, E. In cancer, the extracellular matrix (ECM), a noncellular component of the TME, becomes highly dysregulated, with matrix protein deposition and excessive cross-linking causing matrix stiffening [14]. This increased stiffness is the most notable and recognized mechanical abnormality in solid tumors [15]. Although stiffness is postulated to regulate the stem-like phenotype of HCC cells [16], these associated studies treated HCC or fibrotic liver as tissues with a homogenous increase in stiffness [17] without considering the stiffness heterogeneity in the TME. Given the high heterogeneity of HCC, it first manifests in each patient, it then develops in different tumor nodules of the same patient, and finally in different portions of the same tumor nodules [4]. There is reason to believe that the physical microenvironment of HCC is heterogeneous, however, few studies on this aspect have been conducted.

Mechanotransduction is the mechanism by which cells adapt to the environment by converting mechanical signals from the microenvironment into biochemical signals [18]. Integrins are heterodimeric transmembrane receptors composed of α- and β-subunits that mediate cell adhesion and convey mechanical and chemical signals to the cell interior. Many integrin complexes can sense matrix stiffness [19]. The yes-associated protein (YAP), a transcriptional coactivator of the Hippo pathway, has recently been shown to be a sensor and mediator of mechanical signaling in the ECM. YAP senses changes in cytoskeletal tension [20] and regulates gene transcription mainly by binding to TEA domain family 1–4 (TEAD 1–4) after nuclear translocation [21]. In adult tissues, nuclear YAP is commonly found at sites where somatic stem or progenitor cells are enriched. Additionally, YAP is crucial for tissue repair in vivo and for the growth of organ-specific stem cells as organoids in vitro [18]. Importantly, YAP is commonly overexpressed in murine and human HCC and is associated with adverse outcomes [22].

Here, we aimed to evaluate matrix stiffness heterogeneity in HCC tissues. Our evaluation combined the heterogeneity of HCC cells and the stiffness heterogeneity of the TME, thus, demonstrating the regulatory effect of the TME on stem-like phenotypic changes via mechanotransduction.

## Methods

### HCC samples from patients

Tissue samples were collected from patients with HCC (n = 3) who underwent curative resection at the General Surgery Department, First Hospital of Lanzhou University. Three different regions of the maximum cross-section of HCC tissue were randomly selected. Each sample was cut into two pieces, one of which was immediately transferred to pre-cooled (4 ℃) phosphate-buffered saline (PBS) buffer containing 1% protease inhibitor cocktail (HY-K0010, MCE), while the other was immersed in 4% paraformaldehyde (P1110, Solarbio) for subsequent analyses. This project was approved by the First Hospital of Lanzhou University Ethics Committee (Number: LDYYLL-2021-473). Informed consent was obtained from all patients in accordance with institutional guidelines.

### Histology and immunohistochemistry

Paraffin-embedded tissue samples were cut into 5-μm thick sections, deparaffinized with xylene, and dehydrated using graded alcohol washes. Antigen retrieval was performed for all sections by heating in a microwave oven, and endogenous peroxidase activity was blocked with 3% H_2_O_2_ solution. After 1 h of serum blocking, anti-NANOG (sc-293121, Santa Cruz) or anti-OCT4 (sc-5279, Santa Cruz) antibodies were added to samples and incubated overnight at 4 ℃. The samples were then incubated with a secondary antibody, and then a chromogenic agent (DA1016, Solarbio) was added. In addition, human HCC tissues were stained with Sirius Red. Immunohistochemistry results were analyzed using ImageJ software, and the final results were presented using the average optical density.

### Stiffness measurement

Young's modulus was applied to represent the elastic modulus and characterize the strength of the stiffness. Young’s modulus was measured by atomic force microscopy (AFM) with Nanowizard III (JPK, Germany) in the force spectroscopy mode. For tissue samples, borosilicate glass beads (20 μm diameter) were attached to pyramidal cantilevers (NanoAndMore, USA) with a spring constant of 0.08 N/m. Fresh tissue samples were embedded with OCT (4583, SAKURA) and sliced into 100-μm slices using a Microtome Cryostat (Leica, CM1950). The slices were then placed on adhesive glass coverslips, placed in a 35-mm Petri dish, and approximately 2 ml of pre-cooled (4 ℃) PBS buffer containing 1% protease inhibitor cocktail (HY-K0010, MCE) was added. Measurements were performed immediately. For each sample, indentation tests were performed to generate at least 15 force curves across six 100 × 100 μm^2^ regions. Young’s modulus was calculated using AFM software by fitting the Hertz contact model to the acquired force curves [23].

### Cell cultures

HCCLM3 and Huh7 human HCC cell lines were kindly provided by the Key Laboratory of Biotherapy and Regenerative Medicine (Gansu, China). Cells were cultured in Dulbecco's Modified Eagle Medium (DMEM; C11995500BT, Gibco) supplemented with 10% fetal bovine serum (AB-FBS-1050S, ABW) and 1% penicillin–streptomycin solution (03-031-1B, BI) at 37 °C in a humidified incubator containing 5% CO_2_. Cells were identified using short tandem repeat DNA analysis.

### Preparation of mechanically tunable polyacrylamide gel

Polyacrylamide (PA) hydrogel with tunable stiffness was prepared according to the method described by Tse and Engler [24]. In brief, 500 μl of 0.1 M NaOH (S835850, Macklin) solution was added to round glass coverslips with a diameter of 25 mm and dried in an oven at 80 ℃. Another 500 μl of distilled H_2_O (dH_2_O) was added, dried, and repeated until a uniform NaOH coating was formed on the coverslip surface. 3-Aminopropyltriethoxysilane (A7440, Solarbio) (300 µl) was spread across each coverslip; after 5 min, the coverslips were extensively washed in dH_2_O and then soaked in 0.5% glutaraldehyde (G810413, Macklin) in PBS buffer for 30 min, and air dried for later use. The glass slides were then immersed in dimethyldichlorosilane (D806824, Macklin) for 5 min. Acrylamide (A800656, Macklin) and bis-acrylamide (N813086, Macklin) solutions (Table 1), 1:100 volume of AP (AR1166, Boster), and 1:1000 volume of TEMED (AR1165, Boster) were mixed. The gel mixture (25 µl) was quickly pipetted onto the slides, and inverted coverslips were carefully placed (treated side down) onto the gel droplet. The gel was allowed to polymerize for 5–10 min. The bottom glass slide was removed, and the top coverslip-gel composite was placed in a Petri dish. The mixture was rinsed twice with dH_2_O to remove unpolymerized acrylamide. The dH_2_O was removed, and 800 μl of 0.2 mg/ml sulfo-SANPAH (A35395, Pierce) solution was added to the gel surface. The reaction was carried out for 15 min under UV light. Rinsing was performed twice with 2 ml of 50 mM HEPES solution. Collagen I (354,236, Corning) HEPES solution (1 ml 0.1 mg/ml) was added and incubated overnight at 4 ℃. The cells were rinsed twice with PBS and placed under UV light for 30 min before culturing.

**Table 1:** Expected modulus of elasticity after polymerization of relative acrylamide and bis-acrylamide concentrations

Acrylamide %	Bis-acrylamide %	Acrylamide from 40% stock solution (ml)	Bis-acrylamide from 2% stock solution (ml)	Water (ml)	E ± SD (kPa)
3	0.1	0.75	0.5	8.75	1.10 ± 0.34
5	0.15	1.25	0.75	8	4.47 ± 1.19
10	0.1	2.5	0.5	7	10.61

### Immunofluorescence

Cells cultured on hydrogel with different stiffness were fixed in 4% paraformaldehyde (P1110, Solarbio) for 30 min and permeabilized with 0.2% Triton X-100 (T8200, Solarbio) for 20 min. The cells were then blocked with 10% goat serum (AR1009, Boster) for 1 h and incubated with anti-Ki67 antibody (27309-1-AP, Proteintech) or anti-YAP antibody (13,584–1-AP, Proteintech) overnight at 4 ℃. Subsequently, the cells were incubated with CoraLite488-conjugated secondary antibody (SA00013-2, Proteintech) or rhodamine-conjugated secondary antibody (SA00007-2, Proteintech) for 1.5 h in the dark. F-actin was stained with rhodamine-phalloidin (CA1610, Solarbio). Nuclei were counterstained with 2-(4-amidinophenyl)-6-indolecarbamidine dihydrochloride (DAPI) (AR1176, Boster), and images were captured by fluorescence microscopy (Olympus, IX73). For tissue sections, after deparaffinization and dehydration, the remaining protocol was the same as that for cells.

### Real-time PCR

Total RNA was extracted using TRIzol reagent (9108, Takara) and reverse-transcribed using the PrimeScript RT Reagent Kit (RR047A, Takara). Quantitative PCR was performed using TB Green premix Ex Taq (RR820A, Takara) on a Real-Time PCR Detection System (Bio-Rad, CFX96). Relative mRNA expression was analyzed and normalized to that of *GAPDH*. All reactions were performed in triplicate, and at least three independent experiments were performed. The primer sequences are summarized in Table 2.

**Table 2:** Primers used for real-time PCR

Primer name	Sequence 5′ − 3′
*NANOG*	Forward: AGTCCCAAAGGCAAACAACCCACTTC Reverse: TGCTGGAGGCTGAGGTATTTCTGTCTC
*OCT4*	Forward: GCAGCGACTATGCACAACGA Reverse: AGCCCAGAGTGGTGACGGA
*YAP*	Forward: AACTGCTTCGGCAGGCAAT Reverse: CATCCTGCTCCAGTGTTGGT
*CTGF*	Forward: ACCGACTGGAAGACAGTTTG Reverse: CCAGGTCAGCTTCGCAAGG
*ANKRD*	Forward: GCCCAGATCGAATTCCGTGA Reverse: CGCTGTGCTGAGCAACTTATC
*GAPDH*	Forward: AGAAGGCTGGGGCTCATTTG Reverse: AGGGGCCATCCACAGTCTTC

### Western blotting

Total protein was extracted using RIPA buffer (AR0102, Boster) supplemented with a protease inhibitor cocktail (HY-K0010, MCE) and phosphatase inhibitor (AR1183, Boster). The concentration of extracted proteins was measured using a BCA Protein Assay Kit (PC0020, Solarbio), and equal amounts of extracted proteins were loaded onto SDS-PAGE. The size-separated proteins were transferred to polyvinylidene fluoride (PVDF) membranes (IPVH00010, Millipore) for blotting. After blocking with 5% BSA blocking buffer (SW3015, Solarbio), membranes were incubated overnight at 4 °C with the following specific primary antibodies: anti-YAP antibody (13584-1-AP, Proteintech), anti-phosphorylated YAP antibody (Ser127) (13008 T, Cell Signaling Technology), anti-NANOG antibody (sc-293121, Santa Cruz), anti-OCT4 antibody (sc-5279, Santa Cruz), and anti-GAPDH antibody (10494-1-AP, Proteintech). Following washing, membranes were incubated for 1 h with horseradish peroxidase-conjugated secondary antibodies (SA00001-2, Proteintech). Protein expression was detected using ECL western blotting substrate (PE0010, Solarbio), and the membranes were imaged using a membrane imaging system (Clinx, ChemiScope S6).

### Flow cytometry

HCC cells cultured on hydrogel with different stiffness were collected and washed twice with pre-cooled (4 ℃) PBS. A PE-conjugated anti-human CD133 antibody (394,004, Biolegend) was used for surface marker analysis. For apoptosis testing, cells were stained using an apoptosis kit (40302ES20, Yeasen), according to the manufacturer’s instructions. Flow cytometry was performed using a flow cytometer (Beckman, CytoFLEX) with software.

### Subcutaneous tumorigenesis model of HCC cells mixed with hydrogel of different stiffness

Twenty-four four-week-old male BALB/c nude mice were purchased from the Gempharmatech company (Jiangsu, China). HCCLM3 cells (3 × 10^6^) were mixed with VitroGel (TWG001, TheWell) at various concentrations. VitroGel is a xeno-free, tunable hydrogel that can be adjusted to 30–12,000 Pa by changing the hydrogel concentration with the dilution solution. The dilution ratios are listed in Table 3. The mixture was subcutaneously injected into the upper right flank of mice. Subcutaneous tumor formation was observed after 7 days. The stiff + verteporfin group was intraperitoneally injected with 50 mg/kg/day verteporfin for 7 days, and the remaining nude mice were fed normally. Our experiment conforms to the NIH Guide for Care and Use of Laboratory Animals. All animal experimental protocols were approved by the First Hospital of Lanzhou University Ethics Committee (Number: LDYYLL-2021-473).

**Table 3:** The corresponding Young’s modulus of VitroGel at different dilution concentrations

Dilution ratio	VitroGel (ml)	Dilution solution (ml)	Cell suspension (ml)	Young’s modulus (Pa)
1:0	2	0	0.5	12,000
1:1	2	2	1	3600–6000
1:3	1	3	1	600–1500

### Statistical analysis

GraphPad Prism 8 was used for statistical analyses. The experimental data were presented as the mean ± standard deviation (SD) and were analyzed using Student’s *t*-test. P values are represented as asterisks (*) on graphs (*P < 0.05; **P < 0.01; ***P < 0.001).

## Results

### HCC tissue has heterogeneous matrix stiffness

**Fig. 1:**
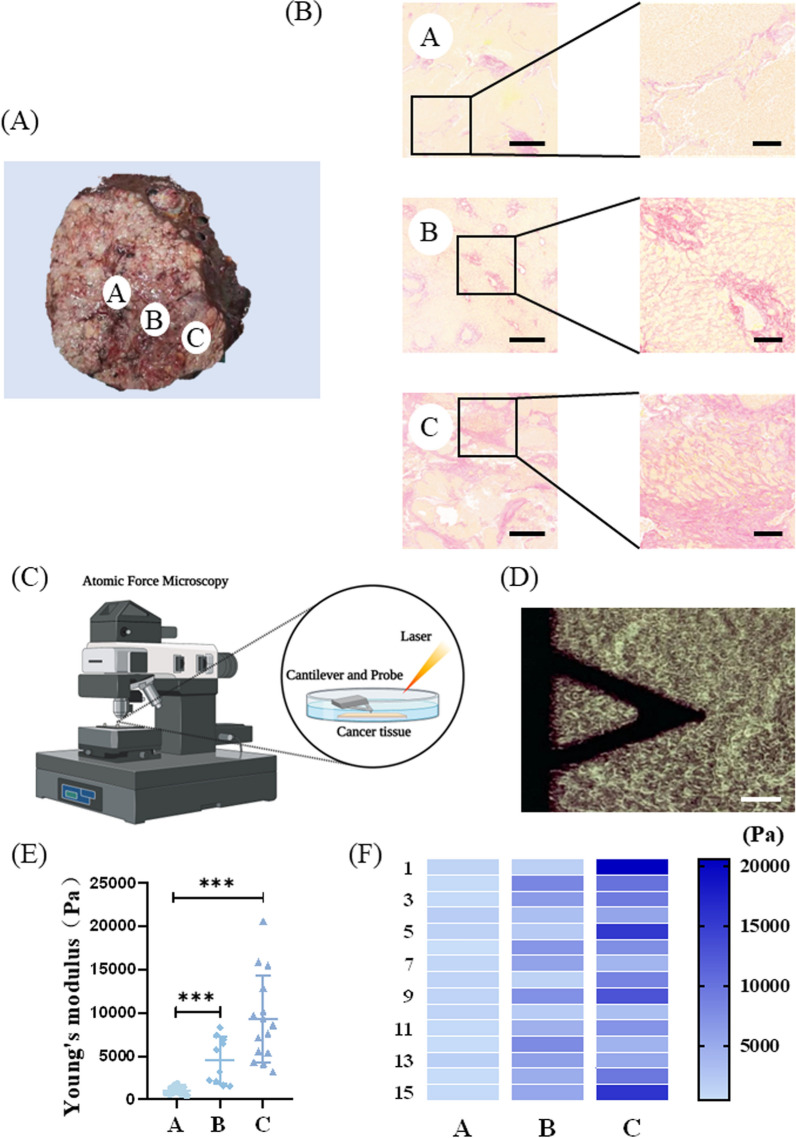
Stiffness measurement of hepatocellular carcinoma (HCC) tissue. **A**Three sites on the cross-section of HCC specimen were randomly selected and labeled as groups A, B, and C. **B** Sirius Red staining in the A, B, and C groups. Scale bar = 500 μm (left). Scale bar = 100 μm (right). **C** Schematic diagram of atomic force microscopy (AFM) analysis of tissue stiffness (Young’s modulus). **D** AFM probe under the microscope. Scale bar = 100 μm. **E** Young's modulus testing results of A, B, and C groups. **F** Young's modulus testing results within each group

To assess the stiffness heterogeneity in HCC tissues, we collected surgical specimens from patients with HCC and randomly selected three tissue samples at the maximum cross-section of the specimens, which were labeled as A, B, and C groups (Fig. 1A). Sirius Red staining was used to evaluate collagen deposition, and AFM was used to determine the local stiffness (Young’s modulus) of the tissue. Sirius Red staining showed that the collagen content in different parts of the tumor tissue differed significantly. Group A had a small amount of collagen, and group B had a medium amount of collagen, whereas group C contained a large number of disordered collagen fibers (Fig. 1B). Further, with AFM (Fig. 1C, D) detection, the Young’s modulus of the tissues was found to be significantly different: group A, E = 1051.61 ± 434.27 Pa; group B, E = 4540.35 ± 2666.98 Pa; group C, E = 9307.37 ± 4989.91 Pa (Fig. 1E). Additionally, the increase in stiffness was consistent with the amount of collagen in the tissue. Similar to the results of previous studies, tissue stiffness largely depended on the amount of collagen deposition and cross-linking [25, 26]. Furthermore, the difference in Young’s modulus was not only reflected in the different sampling sites but also at the same sampling site (Fig. 1F).

### HCC cells exhibit different cellular and cytoskeletal morphology on hydrogel with varying stiffness

**Fig. 2:**
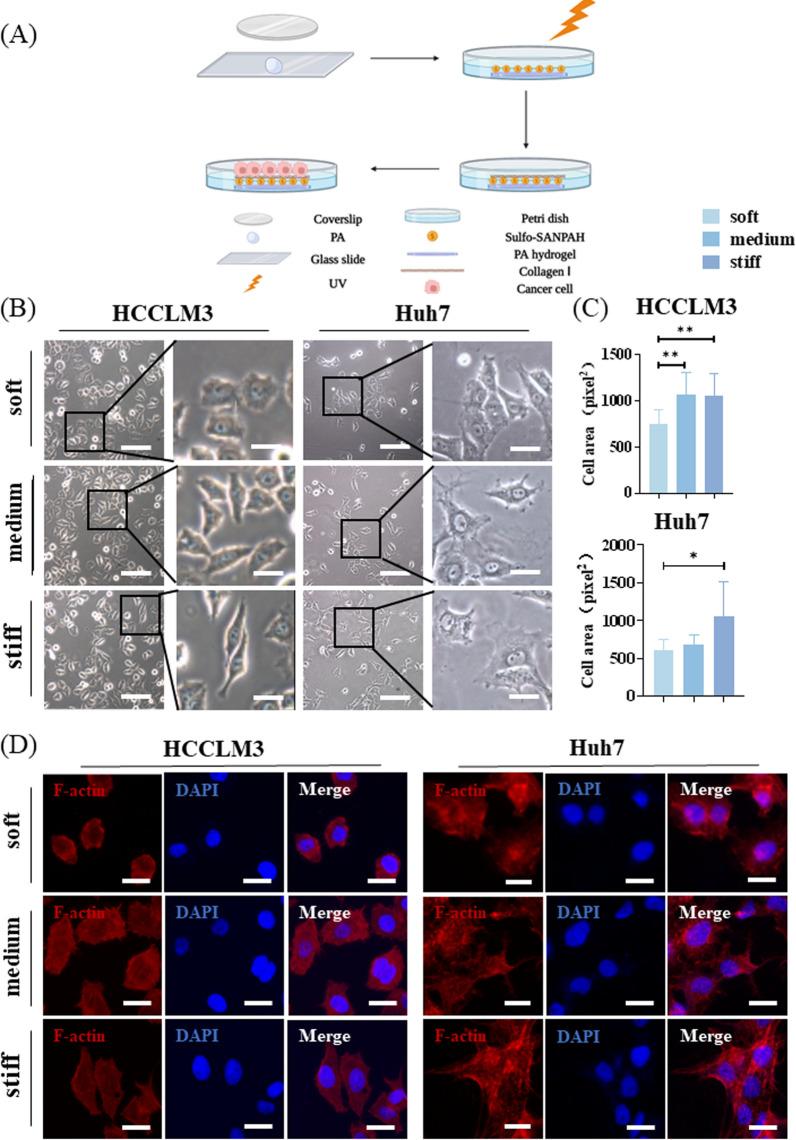
Cellular and cytoskeletal morphology of hepatocellular carcinoma (HCC) cells on polyacrylamide (PA) hydrogel with different stiffness. **A** Schematic diagram of PA hydrogel preparation. **B** Morphology of HCCLM3 and Huh7 cells adhering to hydrogel with different stiffness. Scale bar = 100 μm (left). Scale bar = 20 μm (right). **C** Statistical analysis of cell surface area. **D** Cytoskeleton of HCCLM3 and Huh7 cells on hydrogel with different stiffness. Scale bar = 20 μm

Next, we used collagen I-coated PA hydrogel with adjustable stiffness to simulate the differential stiffness in the tissues (Fig. 2A). PA hydrogel exhibits good biocompatibility and stability. Based on the AFM analysis of HCC tissues, the average stiffness of the different regions (A: 1051.61 Pa, B: 4540.35 Pa, and C: 9307.37 Pa) was used to design the hydrogel. We adjusted the stiffness of the hydrogel by changing the ratio of acrylamide and bis-acrylamide (according to the ratio in the previous literature [24]), and the final stiffness values of the hydrogel were 1.10 ± 0.34 kPa, 4.47 ± 1.19 kPa, and 10.61 kPa (Table 1), represented as soft, medium, and stiff, respectively. The HCC cell lines, HCCLM3 and Huh7, exhibited different cell morphologies on hydrogel with different stiffness. That is, the cell morphology was relatively round on soft hydrogel, while on medium and stiff hydrogel, the cell morphology became gradually elongated and extended. The analysis showed that cells on medium and stiff hydrogel had greater surface areas (Fig. 2B, C). Actin filaments play an important role in mechanosensing [27]. After cytoskeleton staining with rhodamine-labeled phalloidin, we observed that cells on medium and stiff hydrogel had clearer stress fibers and actin filament networks compared with those on the soft hydrogel (Fig. 2D).

### HCC stem-like cell phenotype, drug resistance, and proliferative ability in response to different stiffness

**Fig. 3:**
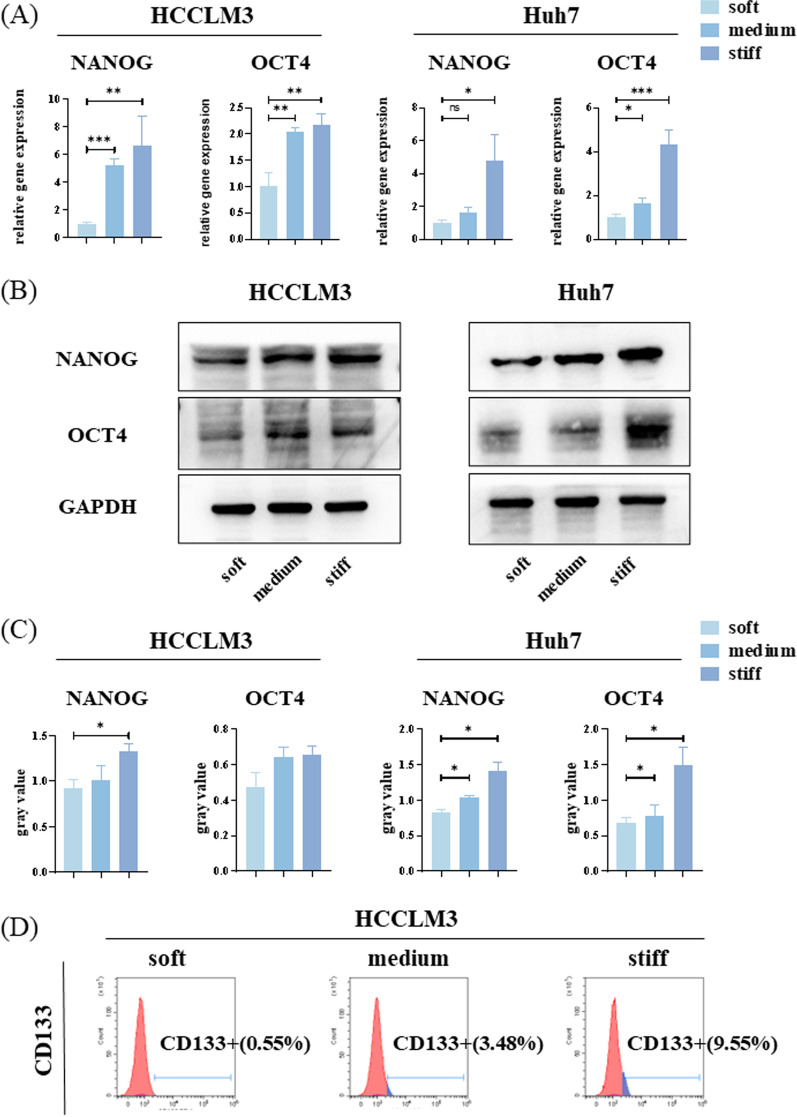
Expression of stem-like phenotypes of hepatocellular carcinoma (HCC) cells on polyacrylamide (PA) hydrogel with different stiffness. **A** Relative mRNA expression levels of *NANOG* and *OCT4* in HCCLM3 and Huh7 cells on hydrogel with different stiffness. **B** Western blot (WB) for NANOG and OCT4 protein abundance in HCCLM3 and Huh7 cells on hydrogel with different stiffness. **C** Relative NANOG and OCT4 protein abundance was measured by quantifying band density using ImageJ software. After normalization to GAPDH protein expression for each sample, the semi-quantitate results were obtained as a ratio. **D** CD133-positive HCCLM3 cells cultured on hydrogel with different stiffness were estimated by flow cytometry

HCCLM3 and Huh7 cells exhibited different stem-like phenotypes on hydrogel with different stiffness. CSC maintenance is regulated by various transcription factors, including NANOG and OCT4 [28]. Differences were detected in the mRNA and protein levels of NANOG and OCT4 based on the hydrogel, from soft to stiff. Compared with the soft hydrogel, the NANOG and OCT4 expression increased in cells on the stiff hydrogel (Fig. 3A, B, C). CD133 is a transmembrane glycoprotein expressed in adult stem cells. Ma et al*.* [29] first reported CD133 as a marker of hepatic CSCs. Flow cytometry analysis showed that the proportion of CD133-positive cells exhibited the same trend as that of NANOG and OCT4 expression, gradually increasing from soft to stiff hydrogel (Fig. 3D, Additional file 1: Fig. S1A).

**Fig. 4:**
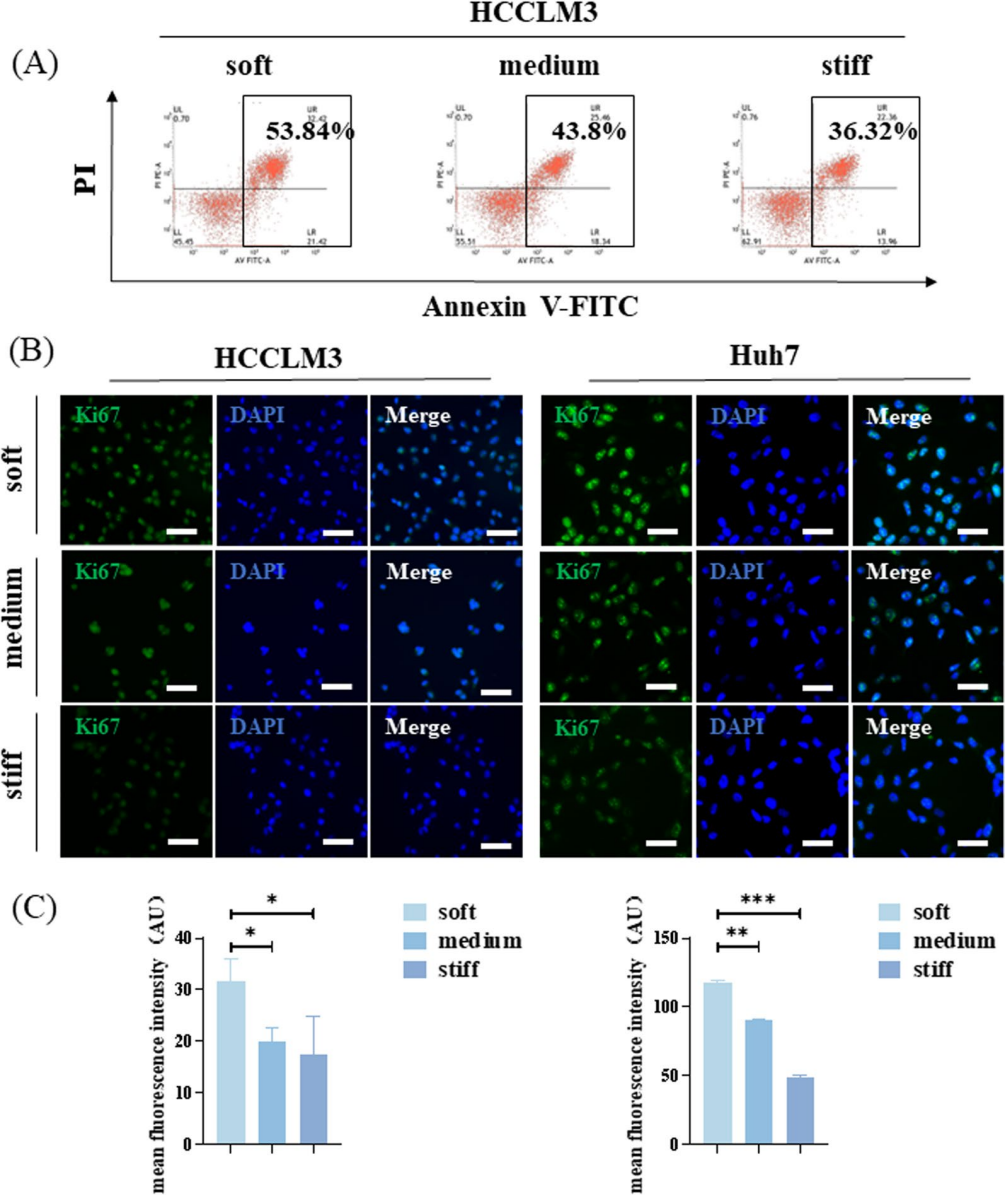
Drug resistance and proliferation ability of hepatocellular carcinoma (HCC) cells on polyacrylamide (PA) hydrogel with different stiffness. **A** Apoptotic HCCLM3 cells on hydrogel with different stiffness treated with sorafenib. **B** Representative immunofluorescence images of Ki67 (green) and 4′,6-diamidino-2-phenylindole (DAPI) (blue) in HCCLM3 and Huh7 cells on hydrogel with different stiffness. Scale bar = 100 μm. **C** Ki67 fluorescence intensity was quantified using ImageJ software

Considering that the stemness of cancer cells is closely related to drug resistance, we treated cells cultured on hydrogel with different stiffness with 10 μM sorafenib for 24 h and then carried out apoptotic cell analysis. The proportion of apoptotic cells on the soft hydrogel was the largest, whereas that on the stiff hydrogel was the smallest (Fig. 4A, Additional file 1: Fig. S1B). Immunofluorescence analysis of proliferation-related protein Ki67 showed that the fluorescence intensity of cells on soft hydrogel was significantly higher than that on medium and stiff hydrogel (Fig. 4B, C).

### Increased matrix stiffness regulates cancer stem-like cell phenotype via the integrin–YAP pathway

**Fig. 5:**
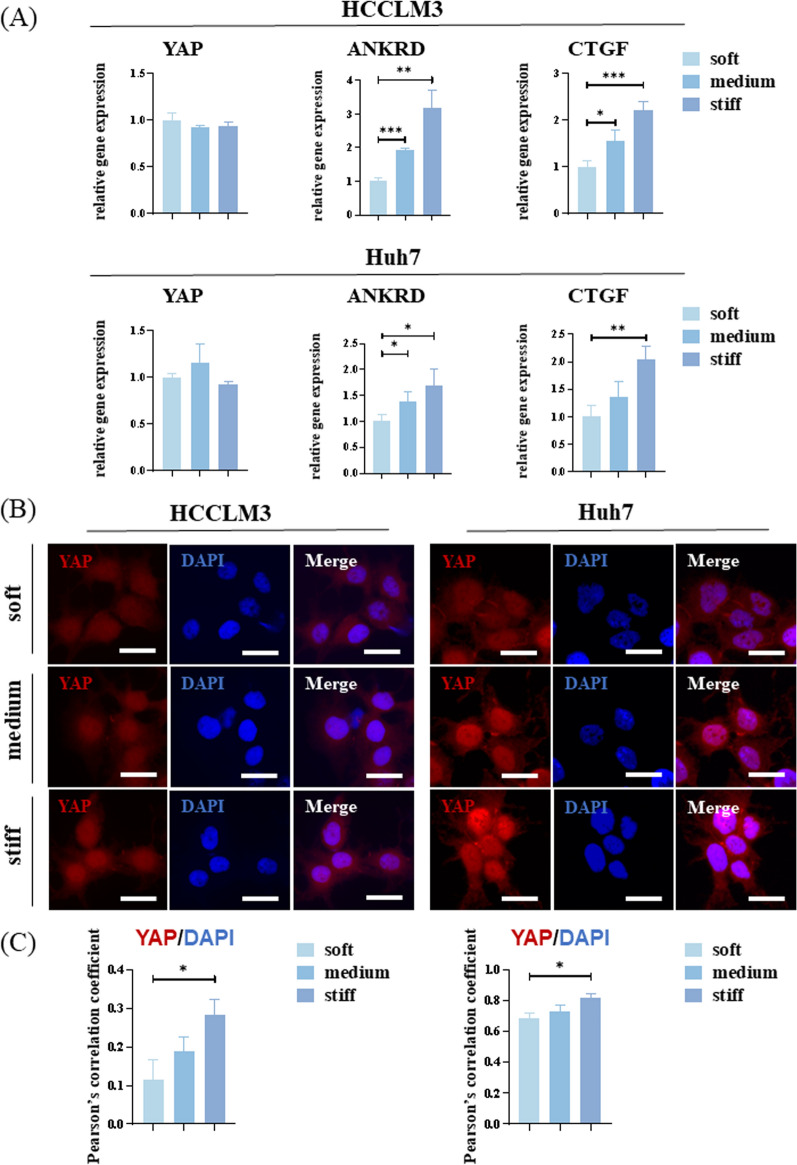
Yes-associated protein (YAP) plays an important role in mechanotransduction. **A** Relative mRNA expression levels of *YAP* and YAP target genes, *ANKRD* and *CTGF*, in HCCLM3 and Huh7 cells on hydrogel with different stiffness. **B** Representative immunofluorescence images of YAP (red) and 4′,6-diamidino-2-phenylindole (DAPI) (blue) in HCCLM3 and Huh7 cells on hydrogel with different stiffness. Scale bar = 20 μm. **C** Quantification of YAP:DAPI nuclear co-localization is represented by Pearson’s correlation coefficient

YAP is a transcriptional coactivator that shuttles from the cytoplasm to the nucleus through dephosphorylation and binds to TEAD and other transcription factors to promote gene expression. YAP is also a transducer of cellular structures, including polarity, morphology, and cytoskeletal structure, and it can reprogram non-stem cancer cells into cells with CSC attributes [30, 31]. To explore the role of YAP in stiffness and cancer cell stemness, PCR analysis was performed. No significant difference in the mRNA expression of *YAP* was observed when cells were cultured on hydrogel with different stiffness. However, *CTGF* and *ANKRD* expression, which are typical downstream genes of YAP, increased significantly with an increase in stiffness (Fig. 5A).

**Fig. 6:**
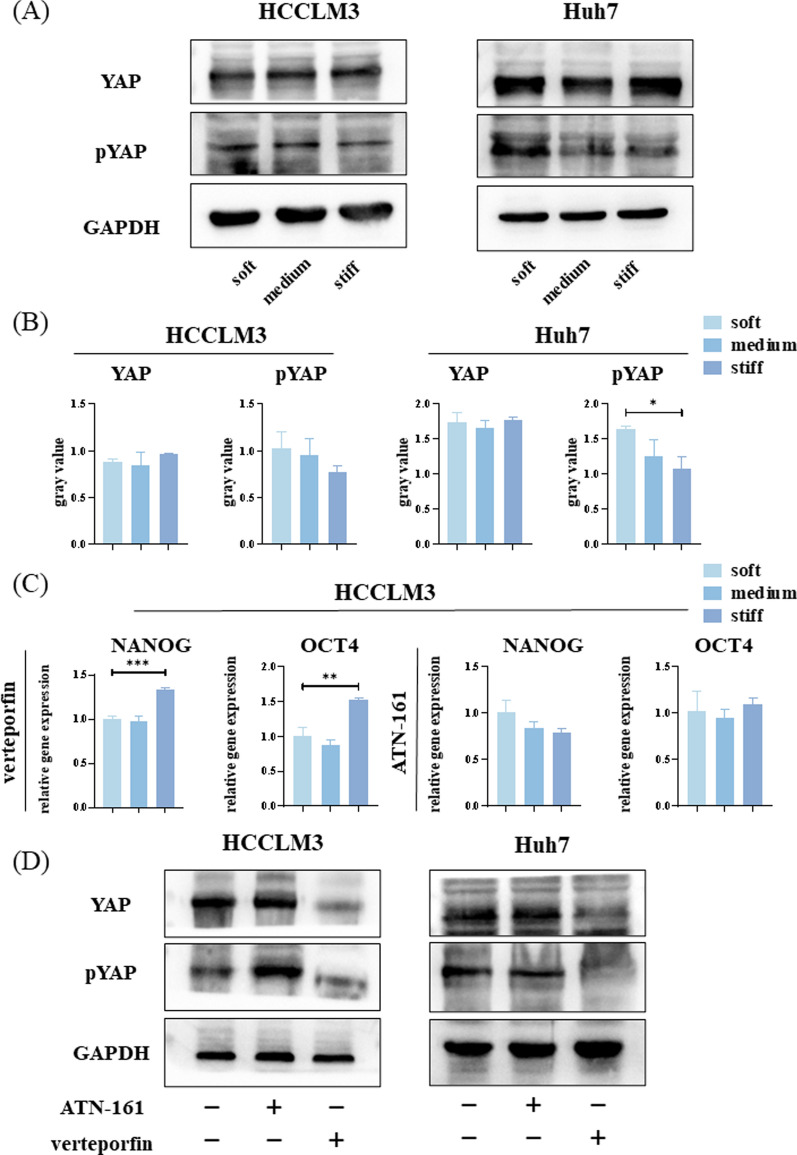
The integrin–yes-associated protein (YAP) pathway conducts mechanical signals into hepatocellular carcinoma (HCC) cells to induce differences in stemness expression. **A** Western blot (WB) analysis of the abundance of YAP and pYAP in HCCLM3 and Huh7 cells on hydrogel with different stiffness. **B** Relative YAP and pYAP protein abundance determined by quantifying band density with ImageJ software. After normalization to GAPDH protein expression for each sample, the semi-quantitate results were obtained as a ratio. **C** Relative mRNA expression levels of *NANOG* and *OCT4* in HCCLM3 cells on hydrogel with different stiffness treated with verteporfin or ATN-161. **D** WB analysis of YAP and pYAP abundance in HCCLM3 and Huh7 cells on stiff hydrogel treated with verteporfin or ATN-161

Considering YAP function, we examined its subcellular localization. Immunofluorescence results indicate that YAP aggregation in the nucleus increased with increasing stiffness (Fig. 5B, C). Subsequent WB results confirmed that there was no significant difference in the total amount of YAP among the three groups. However, the phosphorylated YAP content decreased with increasing stiffness, indicating that the number of activated YAP increased (Fig. 6A, B).

Verteporfin is a YAP inhibitor that inhibits the binding of YAP to TEAD, thereby inhibiting its transcriptional activity. Hence, HCCLM3 cells cultured on hydrogel with different stiffness were treated with verteporfin (1 μg/ml) for 24 h, and the mRNA expression of *NANOG* and *OCT4* was analyzed. *NANOG* and *OCT4* mRNA expression remained higher in cells on stiff hydrogel, however, the differences were notably smaller than those without the inhibitor. Moreover, the ratio of *NANOG* expression on stiff hydrogel and soft hydrogel decreased from approximately 6.7-fold to 1.3-fold, while that of *OCT4* expression decreased from approximately 2.0-fold to 1.6-fold.

Integrins are cell surface proteins that sense the mechanical characteristics of the microenvironment [19]. ATN-161 (Ac-PHSCN-NH2) is a small peptide antagonist of several integrins, including integrin-α5β1. Hence, HCCLM3 cells cultured on hydrogel with different stiffness were treated with ATN-161 (10 μmol/ml) for 24 h and the resulting mRNA expression was analyzed. No significant differences were observed in *NANOG* nor *OCT4* mRNA expression among the three groups. However, the *NANOG* expression was lower in the medium and stiff groups compared with the soft group (Fig. 6C).

These experiments were then repeated in Huh7 cells and the differences in the expressions of *NANOG* and *OCT4* mRNA following verteporfin treatment were similar to that in HCCLM3 cells. Moreover, no significant difference was detected in *NANOG* expression following treatment with ATN-161; whereas OCT4 expression was inhibited to a certain extent (Additional file 2: Fig. S2).

To verify the effectiveness of the inhibitors, ATN-161 and verteporfin were added to HCCLM3 and Huh7 cells cultured on stiff hydrogel for 24 h. WB analyses suggested that there was no significant difference in YAP abundance between the stiff and stiff + ATN-161 groups, whereas it was significantly reduced in the stiff + verteporfin group. Moreover, the abundance of pYAP in the stiff group was lower than that in the stiff + ATN-161 group, and was lower yet in the stiff + verteporfin group (Fig. 6D).

### Matrix stiffness regulates the stem-like phenotype of HCC cells in vivo

**Fig. 7:**
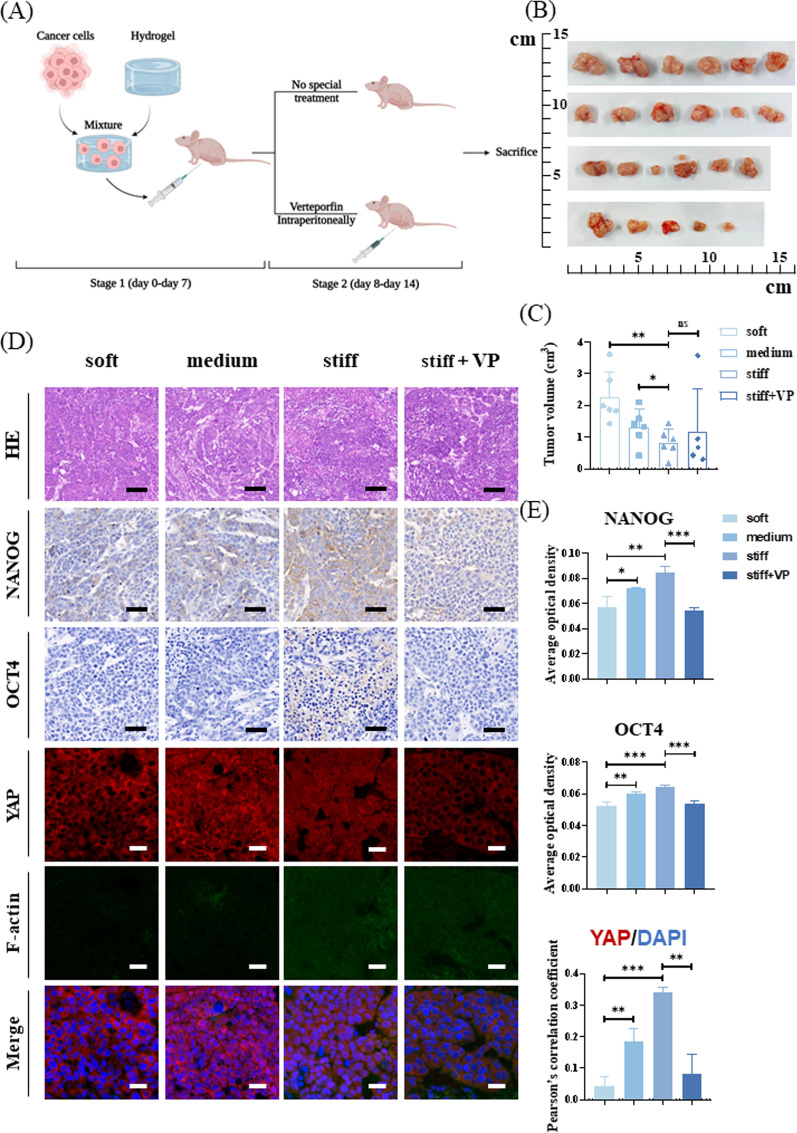
Matrix stiffness regulates stemness expression of hepatocellular carcinoma (HCC) cells in vivo. **A** Flow chart illustrating the establishment of subcutaneous tumors in nude mice with a mixture of HCCLM3 cells and hydrogel. **B** Gross appearance of subcutaneous tumors in nude mice. **C** Analysis and comparison of tumor volume. **D** Immunohistochemical staining and immunofluorescence analysis of NANOG, OCT, YAP, and cytoskeleton. Scale bar = 100 μm (hematoxylin–eosin staining, HE). Scale bar = 50 μm (immunohistochemistry). Scale bar = 20 μm (immunofluorescence). **E** Quantification of NANOG and OCT4 expression is represented by average optical density. Quantification of YAP:DAPI nuclear co-localization is represented by Pearson’s correlation coefficient. (*n* = 6 in soft, medium, stiff groups and *n* = 5 in stiff + VP group)

Finally, we performed subcutaneous tumorigenesis in nude mice to explore whether the cells could reproduce the in vitro experimental results. HCCLM3 cells were mixed with hydrogel of adjustable stiffness and injected subcutaneously into nude mice. After injection, the soft, medium, and stiff groups were fed normal diets. In the stiff + verteporfin (stiff + VP) group, verteporfin (50 mg/kg) was continuously injected intraperitoneally from day 8 to 14 (Fig. 7A). Nude mice were euthanized after 14 days, and subcutaneous tumor tissues were collected. Tumor volumes decreased significantly with an increase in matrix stiffness. However, there were no significant differences between the stiff and stiff + VP groups (Fig. 7B, C). Immunohistochemical staining revealed that the abundances of NANOG and OCT4 proteins increased significantly with increased matrix stiffness. Immunofluorescence results showed that the translocation of YAP into the nucleus increased with increasing matrix stiffness. Moreover, in the soft and medium groups, cytoskeleton morphology was not observed, whereas in the stiff and stiff + VP groups, the cytoskeleton morphology was clear (Fig. 7D, E).

## Discussion

**Fig. 8:**
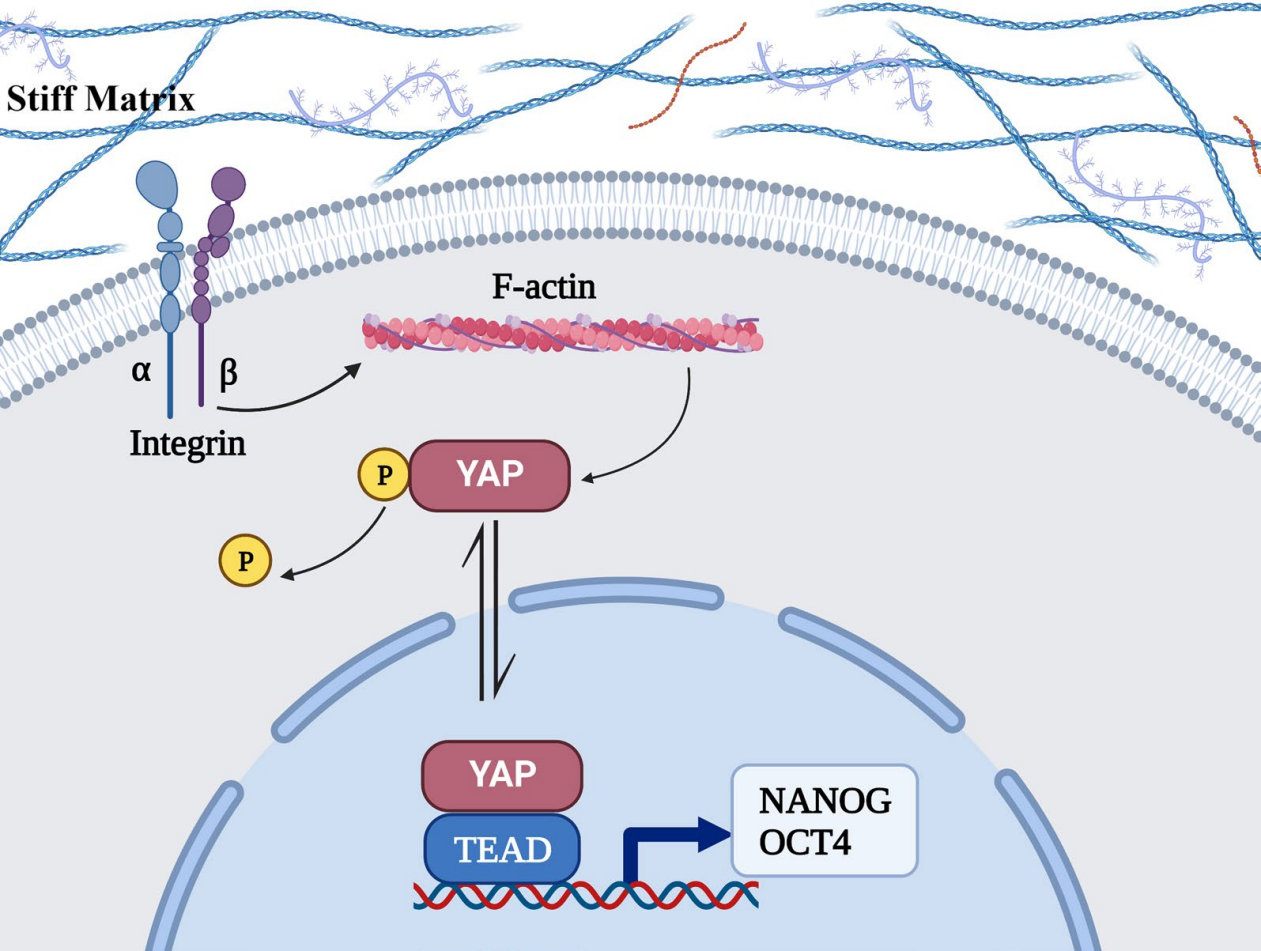
The mechanotransduction pathway. Integrins conduct physical signals into the cell, induce cytoskeleton polymerization, activate YAP and bind to TEAD through nuclear translocation, thereby enhancing the expression of stem-like phenotypes

Here, we described the heterogeneity of matrix stiffness within HCC tissues and how stiffness affects HCC cells via the integrin–YAP pathway to regulate the expression of stem-like phenotypes (Fig. 8).

HCC is a highly heterogeneous tissue. CSCs have been proposed as one of the determining factors that contribute to intratumoral heterogeneity. Stemness traits are acquired via genetic modifications and/or interactions with the TME [10]. According to our results, there is also heterogeneity in the stiffness of the HCC microenvironment, which can directly regulate the expression of stem-like phenotypes via cellular mechanotransduction. According to our analysis of stemness-related HCC markers (NANOG, OCT4 and CD133), OCT4 expression in HCCLM3 cells did not differ significantly between those cultured on medium and high stiffness, which is reflected in both mRNA and protein levels. We postulated that different cells exhibit a range of adaptation to stiffness. That is, there was no significant difference in the induction of stemness expression by stiffness within a certain range.

YAP expression level did not increase with increasing matrix stiffness, while that of its downstream genes, *ANKRD* and *CTGF*, increased. We suggest that YAP might not elicit effects via increased expression but rather through activation. Subsequent WB and immunofluorescence experiments confirmed this hypothesis. A stiffer matrix can promote YAP aggregation in the nucleus by dephosphorylation to increase transcription of NANOG and OCT4. Moreover, application of YAP inhibitor partially prevented the induction of stemness expression by stiff substrates both in vivo and in vitro. Because YAP is not the only factor that regulates stem-like expression, there are other cellular pathways related to stemness.

Integrins can transduce mechanical signals into cells; therefore, we hypothesized that they are upstream regulators of YAP. To test this hypothesis, we treated HCC cells with an integrin inhibitor and found that the expression of stemness-related markers and active YAP was decreased. Moreover, the expression of NANOG on stiff hydrogel was lower than that on soft hydrogel. This may have been caused by the specific integrin inhibitor that was used, ATN-161, as it acts on several integrin isoforms, thereby causing extensive inhibition of the downstream mechanical pathways.

Matrix stiffness can additionally affect drug resistance in HCC cells. Following sorafenib treatment, HCC cells cultured on a stiffer matrix exhibited lowered apoptosis levels, which were positively correlated with the expression of stem-like phenotypes. Matrix stiffening inherently constitutes a mechanical barrier against drug delivery [32] and can also enhance the resistance of the cell itself, thus, complicating chemotherapy. Recently, drugs targeting matrix stiffness have proven effective in experiments and are gradually being applied in clinical settings [33]. However, matrix stiffness can also affect HCC cell proliferation. Here, HCC cells had a stronger proliferative ability when cultured on a soft matrix both in vivo and in vitro. CSC has the ability to self-renew, but this does not equate to greater proliferative ability. Although there is no direct experimental evidence that CSCs undergo cell cycle arrest; in fact, they have been shown to incorporate DNA labels and are therefore often described as slow-cycling cells [5]. Moreover, the drug resistance and responsibility for recurrence make CSCs overlap with dormant cancer cells (non-proliferating cancer cells undergoing G0-G1 cell cycle arrest) [34]. This could lead to another problem: if sorafenib is administered in combination with other treatments that reduce matrix stiffness, the softened matrix will increase the proliferative capacity of HCC cells, thus reducing the therapeutic efficacy. Therefore, drugs that reduce matrix stiffness might not be the best choice for HCC treatments. Indeed, many drugs targeting matrix stiffness have been designed to target CAFs, degrade ECM, or reduce cross-linking [33]; however, their application faces some challenges. For instance, the non-specificity of surface markers of CAFs leads to inaccurate targeting, the role of CAF subsets in tumor promotion and inhibition is not fully understood as well [11]. Moreover, degradation of the ECM may remove the obstacle of cancer invasion [35]. According to our in vivo results, tumor-bearing mice did not show significantly altered tumor size when treated with a YAP inhibitor suggesting that treatments targeting the cellular mechanotransduction pathway may be more effective.

Contrary to our experimental results, previous studies have indicated that the expression of stem-like phenotypes in HCC cells was higher when cultured on soft substrates [16]. This may be due to the application of hydrogel with different stiffness and reactivities with HCC cell lines, which requires further exploration. Meanwhile, in another study, higher matrix stiffness was found to trigger epithelial-mesenchymal transition (EMT) of HCC cells [36] and promote the formation of a pre-metastatic niche [37]. In fact, several studies have shown an association between EMT and the acquisition of stem-like phenotypes [38]; our results are consistent with these experiments.

Here, we aimed to establish a relationship between the heterogeneity of the cancer mechanical microenvironment and the expression of stem-like phenotypes. We observed that HCC cells grown on hydrogel with different stiffness expressed different degrees of stem-like phenotypes. However, the heterogeneity of cancer cells appears during the hierarchical differentiation of CSCs, and we cannot dynamically observe the process of mechanical factors regulating stemness expression owing to the lack of cancer cell markers at different differentiation levels. Therefore, it is challenging to maintain cells at one stage of differentiation to enable analyses such as drug sensitivity. Additionally, our animal experiments used mechanically tunable polysaccharide hydrogel, which differs from previous studies that used components, such as Matrigel or collagen to simulate matrix stiffness. Collagen and other components regulate the mechanical properties via adapting the concentration. Previous experiments have confirmed that the concentration of collagen has a certain impact on the biological behavior of cells [39], as well as other physical properties (such as cell-binding sites, pore size, porosity, and degradability) change [40]. Therefore, these biological materials cannot exclude these effects. Moreover, owing to the high heterogeneity of the TME, simulating it in its entirety in vitro is challenging and will require continued advancement of the associated technology.

## Conclusion

Our experimental results show that the stiffness of the HCC microenvironment is heterogeneous, and the effect of matrix stiffness on cancer cells affects stem-like expression, consequentially forming tiny mechanical niches, leading to the heterogeneity of cells and poor therapeutic effects. In this process, the integrin-YAP pathway conducts mechanical signals to regulate cell function. Hence, targeting cell mechanotransduction pathways may be a new strategy for eradicating drug resistance.

## Supplementary Information

**Additional file 1: Fig. S1. **(A) CD133-positive Huh7 cells cultured on hydrogel with different stiffness were estimated by flow cytometry. (B) Apoptotic Huh7 cells on hydrogel with different stiffness treated with sorafenib.

**Additional file 2**:** Fig. S2. **Relative mRNA expression levels of *NANOG *and *OCT4 *in Huh7 cells on hydrogel with different stiffness treated with verteporfin or ATN-161.

## Data Availability

The datasets analyzed in this study are available from the corresponding author on reasonable request.

## Abbreviations

HCC: Hepatocellular carcinoma
CSC: Cancer stem cell
T-IC: Tumor-initiating cell
TME: Tumor microenvironment
CAF: Cancer-associated fibroblast
ECM: Extracellular matrix
YAP: Yes-associated protein
AFM: Atomic force microscopy
PA: Polyacrylamide
DAPI: 2-(4-Amidinophenyl)-6-indolecarbamidine dihydrochloride
WB: Western blot
EMT: Epithelial-mesenchymal transition
