# Supplementary figures and images for: Heterogeneous matrix stiffness regulates the cancer stem-like cell phenotype in hepatocellular carcinoma
Source: J Transl Med. 2022 Dec 3;20:555. doi: 10.1186/s12967-022-03778-w (PMC9719217; doi:10.1186/s12967-022-03778-w)

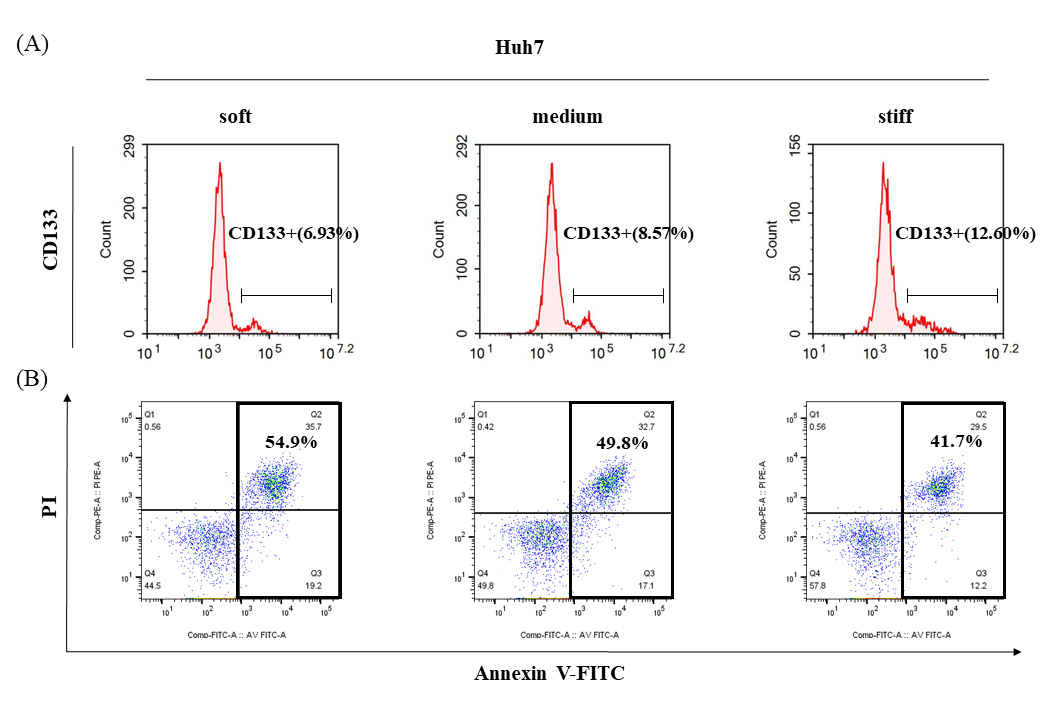

Supplement: Supplementary file 1 — Additional file 1: Fig. S1. (A) CD133-positive Huh7 cells cultured on hydrogel with different stiffness were estimated by flow cytometry. (B) Apoptotic Huh7 cells on hydrogel with different stiffness treated with sorafenib. [file 12967_2022_3778_MOESM1_ESM.tif]

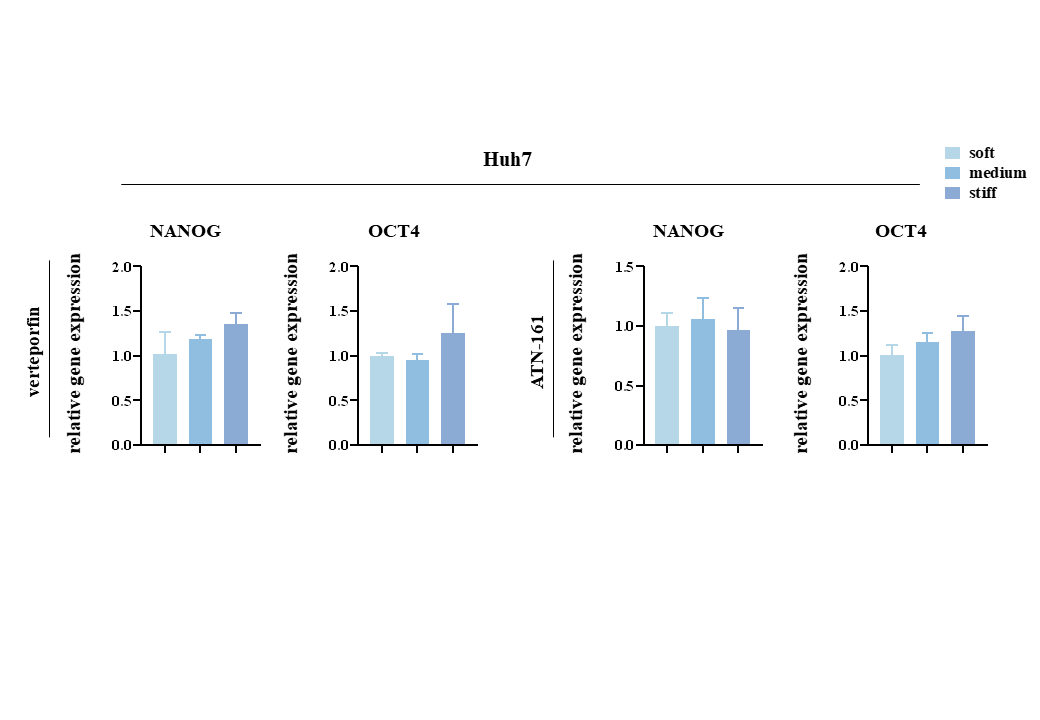

Supplement: Supplementary file 2 — Additional file 2: Fig. S2. Relative mRNA expression levels of NANOG and OCT4 in Huh7 cells on hydrogel with different stiffness treated with verteporfin or ATN-161. [file 12967_2022_3778_MOESM2_ESM.tif]
